# Comparative Evaluation of Traditional and Emerging Glycemic Biomarkers in Diabetes

**DOI:** 10.3390/cimb48090866

**Published:** 2026-08-26

**Authors:** Jeremy Jones, Kyla Frenia, Kathleen C. Garwood, Kunhong Xiao, Leanne T. Labriola

**Affiliations:** 1 Pittsburgh Research Institute, Sewickley, PA 15143, USA; 2 Department of Bioengineering, Swanson School of Engineering, University of Pittsburgh, Pittsburgh, PA 15260, USA; 3 Department of Decision and System Sciences, Saint Joseph’s University, Philadelphia, PA 19131, USA; 4 Department of Biomedical Engineering, College of Engineering, Carnegie Mellon University, Pittsburgh, PA 15213, USA; 5 Center for Proteomics & Artificial Intelligence, Allegheny Health Network Cancer Institute, Pittsburgh, PA 15212, USA; 6 Center for Clinical Mass Spectrometry, Allegheny Health Network Cancer Institute, Pittsburgh, PA 15212, USA

**Keywords:** diabetes, retinopathy, glycated, albumin, biomarker, glucose, tear fluid, diagnostic, monitoring

## Abstract

Diabetes mellitus is a worldwide metabolic disorder that can be debilitating to many if uncontrolled. Biomarkers, such as hemoglobin A1C and glycated albumin, are central to the effort to control diabetes. These each have their own advantages and disadvantages, and there is much ongoing research into development of improved biomarkers and biomarker measurement methods. Continuous glucose monitoring is also widely used to closely monitor diabetes in patients today. Increasingly, however, there has been research focusing on the use of biomarkers in alternative mediums, such as tear fluid or saliva. This review efficiently summarizes the current biomarkers used clinically today, their advantages and disadvantages, and the biomarkers on the horizon that could advance how diabetes is treated in the future.

## 1. Introduction

Diabetes mellitus is a global health crisis that grows in severity year after year, affecting an estimated 537 million adults worldwide in 2021, with projections that could exceed 700 million by 2045 [1]. Uncontrolled diabetes can be debilitating for many individuals, as it often leads to widespread, systemic complications such as retinopathy, nephropathy, and cardiovascular disease. Early diagnosis and close monitoring of this disease is crucial to slowing the progression complications. Several conventional biomarkers are currently used for diabetes diagnosis and monitoring, including hemoglobin A1c (HbA1c), fasting plasma glucose (FPG), oral glucose tolerance test (OGTT), continuous glucose monitoring (CGM), fructosamine, and glycated albumin (GlyAlb), as summarized in Table 1. HbA1c is the most widely used biomarker in diabetes control, but other biomarkers such as CGM and GlyAlb are emerging as new, valuable methods of monitoring blood glucose.

Despite these advances, important gaps remain. The performance of these biomarkers in diagnosing disease, especially in the early stages of the disease or in gestational diabetes, is still under investigation. Simultaneously, interest in minimally invasive diagnostics has increased and other noninvasive biofluids, such as tear fluid, are emerging as a potential alternative to blood-based measurement. In this manuscript, we compare and contrast the three most utilized biomarkers for diabetes (HbA1C, CGM, and GlyAlb) in terms of their diagnostic, prognostic, and monitoring utility, all with the intent of best defining their utility in diabetes management. We also briefly evaluate tears as a diagnostic fluid for diabetic biomarkers, assessing the feasibility and potential that they hold as an alternative source of biomarker information.

This is a narrative review synthesizing evidence on established and emerging glycemic biomarkers in diabetes. Literature was identified through searches of PubMed and Google Scholar using terms including “HbA1c,” “continuous glucose monitoring,” “glycated albumin,” “fructosamine,” “diabetes diagnosis/monitoring,” and “tear fluid biomarkers,” supplemented by manual reference-list screening. Searches were generally limited to the past 15 years, except for landmark trials (e.g., DCCT, UKPDS). Included articles were peer-reviewed, in English, and addressed biomarker diagnostic, prognostic, or monitoring performance in humans; case reports, conference abstracts, and non-English or irrelevant studies were excluded. As this is a narrative review, no systematic protocol (e.g., PRISMA) was followed.

## 2. Hemoglobin A1c

HbA1c is formed through the non-enzymatic glycation of hemoglobin within red blood cells, providing an integrated measure of average glycemia over approximately 2–3 months, corresponding to the lifespan of erythrocytes [2]. It is widely recognized as a cornerstone biomarker for diabetes diagnosis and management, with the American Diabetes Association (ADA) and World Health Organization (WHO) endorsing HbA1c ≥ 6.5% as a diagnostic threshold for diabetes and 5.7–6.4% for prediabetes [3]. HbA1c has also been shown to be a prognostic biomarker for some diabetic complications in both type 1 and type 2 diabetics, as evidenced by landmark trials such as the Diabetes Control and Complications Trial (DCCT) and UK Prospective Diabetes Study (UKPDS), which demonstrated a linear relationship between HbA1c and the risk of microvascular and macrovascular complications, namely retinopathy, nephropathy, and neuropathy [4,5]. In clinical practice, HbA1c remains the most widely used marker for monitoring long-term glycemic control, though it fails to capture short-term fluctuations and glycemic variability that are increasingly recognized as clinically important [2].

Despite its status as the “gold standard,” HbA1c has several notable limitations. Its accuracy is notably compromised in the presence of anemia, particularly iron-deficiency anemia or hemolytic anemia, because it changes red blood cell (RBC) lifespan. Shorter-lived RBCs have less time for hemoglobin glycation, leading to falsely low HbA1c, whereas iron deficiency can sometimes increase glycation rates, causing falsely elevated values [6]. Chronic kidney disease (CKD) can also cause variability in HbA1c readings. CKD can lead to anemia of chronic disease in some cases, which in turn shortens red blood cell lifespan and can lower HbA1c independently of actual glycemia. Additionally, accumulation of carbamylated hemoglobin in CKD patients can interfere with certain assay methods, producing falsely elevated or variable HbA1c readings. Altered erythropoiesis and protein metabolism in CKD further contribute to discrepancies between HbA1c and true average glucose levels, making it less reliable for monitoring glycemic control in this population [7]. Furthermore, hemoglobinopathies, such as sickle cell disease or hemoglobin C variants, alter the structure of hemoglobin, potentially skewing glycation measurements or interfering with assay detection [8]. Variations in glycation rates among different ethnic populations can also result in HbA1c values that do not accurately reflect average glycemia [9]. These mechanisms illustrate why HbA1c may be misleading in certain patient populations and emphasize the value of complementary biomarkers or direct glucose monitoring.

## 3. Continuous Glucose Monitoring (CGM)

CGM measures interstitial glucose levels in real time using subcutaneous sensors, typically providing readings every 1–5 min. Unlike HbA1c, which reflects long-term average glycemia, CGM captures dynamic glucose fluctuations throughout the day [10]. Most CGM systems use enzymatic electrochemical sensors in which glucose oxidase catalyzes the oxidation of glucose in the interstitial fluid, generating an electrical current proportional to glucose concentration that is converted by the device into a glucose reading. These features make CGM a valuable tool for therapeutic monitoring, particularly in patients with type 1 diabetes and insulin-treated type 2 diabetes.

In clinical practice, CGM is implemented using either personal devices worn continuously by patients or professional CGM systems applied intermittently in the clinic to assess glycemic patterns over a defined monitoring period, typically 10–14 days. Data from both modalities are commonly reviewed retrospectively and summarized using the Ambulatory Glucose Profile (AGP), a standardized, single-page report that time-aligns glucose values to a 24 h period and displays median glucose and percentile ranges. This visualization allows clinicians to efficiently identify recurrent hypoglycemia, postprandial hyperglycemia, nocturnal patterns, and overall glycemic variability [11].

CGM-derived metrics used in routine care include mean glucose, time in range (TIR), time below range (TBR), time above range (TAR), and measures of glycemic variability such as standard deviation and coefficient of variation [12]. These metrics complement HbA1c by capturing clinically relevant dimensions of glycemic control not reflected by long-term averages, particularly hypoglycemia burden and glucose excursions. In clinical encounters, CGM data are frequently used to guide insulin dose adjustments, optimize the timing of meals and medications, and provide targeted behavioral feedback to patients [13,14]. CGM platforms also enable the derivation of secondary glycemic indicators, including estimated average glucose and the glucose management indicator (GMI), which approximate HbA1c equivalents based on sensor data. These derived metrics enhance clinical interpretability and facilitate communication between clinicians and patients, particularly as CGM data are increasingly incorporated into quality frameworks and performance reporting [Table 2]. In 2025, NCQA’s HEDIS measure for glycemic status assessment was updated to allow either traditional HbA1c results or CGM-derived GMI to satisfy glycemic control criteria, reflecting broader acceptance of CGM metrics in clinical evaluation [15,16].

Beyond monitoring, accumulating evidence links CGM metrics, particularly TIR, to microvascular complications, supporting their role as clinically meaningful monitoring and prognostic tools, although data linking CGM metrics to long-term macrovascular outcomes remain limited. Despite its clinical utility, CGM interpretation is subject to important limitations. Firstly, measurement of interstitial glucose closely tracks plasma glucose but exhibits a short physiological lag due to glucose diffusion from the vascular compartment into the interstitial space, a delay that becomes most apparent during periods of rapid glucose change such as postprandial excursions or exercise. Additionally, accuracy may decline in hypoglycemic ranges, and artifacts such as sensor compression, signal loss, or data gaps can complicate pattern recognition [17,18]. These limitations highlight the need for clinician familiarity with CGM technology and careful contextual interpretation when integrating CGM data into treatment decisions.

## 4. Glycated Albumin (GlyAlb)

Fructosamine assays were first developed in the 1980s as a practical alternative to HbA1c to offer faster feedback on glycemic control for patients and clinicians. Fructosamine refers to the glycated serum proteins (primarily albumin) and reflects average glycemia over a shorter period of approximately 2 to 3 weeks, corresponding to the half-life of circulating proteins [19]. GlyAlb particularly represents a major fraction of fructosamine and is formed by non-enzymatic glycation of circulating albumin in blood serum. GlyAlb is similar to fructosamine but more specific. It measures isolated albumin rather than all serum proteins, reflecting average glycemia over the prior 2 to 3 weeks.

GlyAlb has shown promise as a clinically relevant diagnostic and prognostic biomarker, with multiple studies demonstrating associations with microvascular and macrovascular outcomes that are comparable to those observed for HbA1c [20,21,22,23,24]. Elevated GlyAlb levels have been linked to increased risks of diabetic retinopathy and nephropathy, as well as coronary heart disease, stroke, heart failure, and all-cause mortality, with effect sizes similar to HbA1c in large population-based cohorts such as ARIC [20]. Several studies provide direct evidence for an association between GlyAlb and diabetic retinopathy, including cross-sectional analyses showing higher GlyAlb levels in individuals with retinopathy compared to those without [21] and longitudinal studies demonstrating that GlyAlb, but not HbA1c, was significantly elevated in patients who developed retinopathy [22]. Importantly, visit-to-visit variability in GlyAlb has also been associated with increased retinopathy risk, whereas HbA1c variability was not, suggesting that GlyAlb may better capture clinically relevant glycemic instability [23]. Consistent with these findings, GlyAlb has shown associations with prevalent retinopathy and incident chronic kidney disease even in individuals without diagnosed diabetes [19] and may provide a more accurate assessment of glycemic exposure than HbA1c in patients with end-stage kidney disease [24].

GlyAlb has both advantages and limitations as a biomarker for diabetes management. Compared with HbA1c, GlyAlb may better reflect postprandial hyperglycemia and short-term glycemic fluctuations since the glycation speed of GlyAlb is ten times faster than HbA1c, offering additional clinical insight in patients with unstable glucose control [25]. Additionally in monitoring, GlyAlb is advantageous in conditions where HbA1c is unreliable, such as anemia, chronic kidney disease, or hemoglobinopathies, although it too is affected by disorders of albumin metabolism, including liver disease, nephrotic syndrome, and thyroid dysfunction [26,27]. It has also been reported that GlyAlb’s role in predicting macrovascular complications such as coronary artery disease may be limited [28]. While not yet widely implemented in clinical practice, GlyAlb represents an emerging biomarker that complements existing tools and may hold particular value in precision diabetes care.

## 5. Future Directions

Despite substantial advances in diabetes biomarker research, several critical gaps remain that present opportunities for future investigation. First, multi-biomarker strategies hold potential to improve how diabetes is monitored and treated. Integration of cumulative glycemic exposure markers (e.g., HbA1c, FBS, GlyAlb) with CGM or OGTT data may enable more comprehensive characterization of glycemic dysregulation across temporal scales. Future research should explore algorithmic approaches that combine long-term markers (HbA1c), intermediate-term markers (glycated albumin, fructosamine), and CGM-derived metrics (e.g., time in range, glycemic variability) to improve prediction of both microvascular and macrovascular complications and to support individualized therapeutic decision-making and real-time treatment adjustment.

The standardization and clinical validation of alternative biomarkers such as GlyAlb and fructosamine are needed. While these markers show promise for short-term glycemic monitoring, particularly in patients with conditions that compromise HbA1c accuracy, standardized cutoffs across diverse populations and assay platforms remain lacking. Large prospective studies evaluating their prognostic and diagnostic performance in early, gestational, and atypical diabetes populations will be crucial, especially given the promising potential of GlyAlb in tear fluid.

Other minimally invasive biofluids (e.g., tear fluid, saliva, sweat) represent an exciting frontier in biomarker development. A recent paper shows that GlyAlb can be reliably measured in tears, suggesting that noninvasive monitoring using tears could be used to more optimally identify and follow diabetes in patients [29]. Further evaluation is needed in larger studies to investigate whether tear-based biomarkers can track glycemic control longitudinally, compare favorably with blood-based measurements, and expand to other markers to best track all aspects of diabetes. Future studies should assess whether tear-derived biomarkers can track glycemic control over time, compare favorably with blood-based measurements, and support composite metrics, such as GlyAlb/HbA1c ratios, that better capture short-term glycemic variability. Expansion to additional biomarkers reflecting systemic inflammation, oxidative stress, and microvascular dysfunction may further enhance risk stratification for diabetes-related complications.

Other studies have shown saliva to hold potential as a noninvasive biofluid for diabetic monitoring, although more robust evidence is needed to support the validity and reliability of saliva as a biofluid. Saliva has been shown to hold some biomarkers that correlate to patients with diabetes [30], and a positive correlation has been shown to link glycemic control and α-2-macroglobulin levels in type 2 diabetics [31]. Other biomarkers in saliva have been correlated to type 2 diabetic patients, including melatonin and pH [32,33]. Interestingly, glucose is detectable in sweat, but at micromolar concentrations only and a variety of other inconsistencies in measurement make sweat a relatively poor biofluid for diabetic monitoring [34].

Advances in sensitive detection platforms, including mass spectrometry and microfluidic immunoassays, will be critical to enabling standardized and clinically scalable measurement of diabetic biomarkers in alternative biofluids. These technologies may also support biomarker-guided intervention studies aimed at preventing disease progression or mitigating complications by enabling earlier risk identification and treatment stratification. Precision medicine approaches that integrate biofluid-derived biomarkers with inflammatory, oxidative, and microvascular signatures alongside genomic, metabolomic, and lifestyle data could ultimately yield predictive models for diabetes onset, progression, and therapeutic response.

Overall, future research in diabetes biomarkers should aim to standardize measurement, integrate multimodal data, exploit noninvasive sample sources, and move toward individualized risk assessment, thereby enhancing both clinical care and patient engagement in disease management.

## 6. Conclusions

HbA1c remains the most established tool for long-term monitoring of blood glucose, but its limitations in cases of certain diseases that impact hemoglobin such as anemia or CKD underscore the importance of complementary measures like glycated albumin and fructosamine. Also, HbA1c tells the story of the past two to three months when measured, which is valuable for diagnosis and prognosis but fail to capture the more subtle day-to-day changes. Although HbA1c has been most utilized by physicians to date, monitoring for glucose variability day-to-day is becoming increasingly important. CGM can be especially valuable as a complement system to HbA1c measurement. However, limitations in the understanding and application of these highly utilized biomarkers emphasize the need to further evaluate alternative biomarkers and measurement modalities to enhance patient access and optimize health outcomes in diabetes care. The use of other noninvasive biofluids such as tear fluid needs to be explored more in the future and may improve the way diabetes is managed and monitored.

## Figures and Tables

**Table 1:** Comparative analysis of glycemic biomarkers.

Biomarker	Temporal Integration	Clinical Utility/Diagnostic Value	Limitations/Confounders
HbA1c	Long-term average glucose (~8–12 weeks)	Diagnosis; monitoring long-term trends; risk stratification for micro- and macrovascular complications	Affected by red blood cell disorders, hemoglobin variants, ethnicity
Continuous Glucose Monitoring (CGM)	Continuous, high-resolution glucose fluctuations	Monitoring glucose variability; detecting hypoglycemia or hyperglycemia patterns; postprandial excursions	Interstitial lag; device errors; cost; patient adherence
Glycated Albumin (GlyAlb)	Intermediate-term glucose exposure (~2–4 weeks)	Monitoring intermediate-term glycemic control; useful when HbA1c is unreliable (anemia, hemoglobinopathies); complement to CGM	Affected by albumin turnover, liver disease, nephrotic syndrome, thyroid disorders
Fasting Plasma Glucose (FPG)	Point-in-time snapshot of fasting glucose	Diagnosis of diabetes; screening	Day-to-day variability; limited postprandial information
Oral Glucose Tolerance Test (OGTT)	Acute, dynamic response to glucose load	Diagnosis of diabetes and impaired glucose tolerance; gestational diabetes assessment	Reproducibility issues; patient inconvenience; pre-test preparation required

**Table 2:** FDA-approved CGM systems (U.S.).

CGM System	Use	FDA Clearance/Status	Coverage (Medicare/Medicaid/Commercial)	HEDIS (Glycemic Assessment)	Clinical Use and Notes
Dexcom G6/G7 (Personal)	Personal (patient-owned real-time CGM)	G6 cleared 2018 (ages ≥ 2); G7 cleared 2022 (ages ≥ 2; iCGM-designated). No fingerstick calibration required.	Medicare covers all insulin-treated beneficiaries; commercial insurers widely cover with Rx; Medicaid varies by state (often follows Medicare criteria).	CGM-derived GMI can substitute for HbA1c in glycemic status measures.	Used for intensive management of Type 1 and insulin-treated Type 2 diabetes (including pediatrics ≥ 2 y); improves A1C and time-in-range. Follow-up (G7) compatible with AID systems (pump integration) and wearables.
Dexcom G6 Pro	Professional (clinic-owned CGM)	Cleared 2019 as the first single-use professional CGM (10-day sensor, ages ≥ 2). Blinded or real-time modes.	Coded as DME/professional service. Medicare and commercial payers cover professional CGM under durable DME or physician services.	Same HEDIS note (CGM GMI can be used).	Short-term CGM applied in clinic (by HCP) to gather glucose data for therapy adjustments. Used for CGM-naïve patients or when retrospective review is needed.
Abbott FreeStyle Libre (personal)	Personal (patient-owned/intermittent scan CGM)	Libre 14-day FDA approved 2018; Libre 2, FDA approved 2020; adds alarms; Libre 3, FDA approved 2022, real-time smartphone CGM. One-point calibration (fingerstick only to verify).	Medicare widely covers Libre systems for insulin-managed diabetes; most commercial plans cover; Medicaid varies by state.	GMI from Libre CGM accepted (same HEDIS).	Used by people with T1D and insulin-requiring T2D (and gestational diabetes) for routine monitoring. Libre 2/3 provide optional real-time alerts. Not FDA-indicated as replacement for fingerstick, but often used adjunctively.
Abbott FreeStyle Libre Pro	Professional (clinic-placed CGM)	FDA approved 2016 for professional use. Disposable 14-day sensor applied in clinic, no patient interaction (blinded).	Covered as a professional CGM service (CPT 95250/95251) by Medicare and commercial payers, given clinical justification.	HEDIS note (GMI use).	Clinic-applied 14-day CGM provides comprehensive glucose trend reports (AGP) to guide therapy. No fingersticks needed. Lower cost alternative to other professional CGMs.
Medtronic Guardian Connect	Personal (mobile CGM)	FDA-cleared 2018 for ages 14–75; uses Guardian Sensor 3 (7-day wear, requires 2 fingerstick calibrations/day). Smartphone app only (no receiver).	Medicare does not cover standalone Guardian Connect (smartphone-only) because no durable receiver. Commercial plans may cover as DME (varies); Medicaid varies by state.	GMI usable.	Mobile real-time CGM (data to CareLink web) with predictive alerts and integrated “Sugar.IQ” AI insights. Suited for T1D and insulin-using T2D patients who want smartphone-based monitoring (Medtronic pump users use pump-integrated CGM).
Medtronic iPro2	Professional (retrospective CGM)	Approved for retrospective CGM (up to 6-day recorder) in mid-2010s.	Covered via professional CGM codes (CPT 95250/95251) by Medicare/commercial when criteria met.	GMI usable.	HCP-applied CGM recorder worn 3–6 days; retrospective data downloaded/printed for treatment planning (used for T2D and T1D without pump).
Senseonics Eversense (Ascensia)	Personal (implantable CGM)	Original Eversense approved 2018 (PMA) for 90-day implant; Eversense 365 (one-year sensor) cleared Sept 2024 for ages ≥ 18.	Medicare covers Eversense (on-body vibration alerts count as durable device); commercial insurers cover as DME (some require prior auth); Medicaid varies.	GMI usable.	Long-term implantable CGM (sensor under skin, removable transmitter). Indicated for T1D and insulin-treated T2D adults. Advantages: up to 90-day or 365-day wear with fewer sensor changes and on-body vibration alarms.

## Data Availability

No new data were created or analyzed in this study. Data sharing is not applicable to this article.

## Abbreviations

The following abbreviations are used in this manuscript:

ADA	American Diabetes Association
ARIC	Atherosclerosis Risk in Communities (study)
CGM	Continuous Glucose Monitoring
CKD	Chronic Kidney Disease
DCCT	Diabetes Control and Complications Trial
GlyAlb	Glycated Albumin
HbA1c	Hemoglobin A1C
HPLC	High Performance Liquid Chromatography
RBC	Red Blood Cell(s)
TBR	Time below range
TIR	Time in range
UKPDS	United Kingdom Prospective Diabetes Study
WHO	World Health Organization
